# Challenging the paradigm of nitrogen cycling: no evidence of *in situ* resource partitioning by coexisting plant species in grasslands of contrasting fertility

**DOI:** 10.1002/ece3.1244

**Published:** 2014-12-23

**Authors:** Anna Wilkinson, Paul W Hill, María V Vaieretti, John F Farrar, Davey L Jones, Richard D Bardgett

**Affiliations:** 1 Lancaster Environment Centre, Lancaster UniversityBailrigg, Lancaster, LA1 4YQ, U.K; 2 Faculty of Life Sciences, Michael Smith Building, The University of ManchesterOxford Road, Manchester, M13 9PT, U.K; 3 School of Environment, Natural Resources and Geography, College of Natural Sciences, Bangor UniversityGwynedd, LL57 2UW, U.K; 4 Instituto Multidisciplinario de Biología Vegetal (IMBIV)Casilla de Correo 495, 5000, Córdoba, Argentina

**Keywords:** Dissolved inorganic nitrogen, dissolved organic nitrogen, grassland productivity, nitrogen cycling, nitrogen partitioning, peptide, soil

## Abstract

In monoculture, certain plant species are able to preferentially utilize different nitrogen (N) forms, both inorganic and organic, including amino acids and peptides, thus forming fundamental niches based on the chemical form of N. Results from field studies, however, are inconsistent: Some showing that coexisting plant species predominantly utilize inorganic N, while others reveal distinct interspecies preferences for different N forms. As a result, the extent to which hypothetical niches are realized in nature remains unclear. Here, we used *in situ* stable isotope tracer techniques to test the idea, in temperate grassland, that niche partitioning of N based on chemical form is related to plant productivity and the relative availability of organic and inorganic N. We also tested *in situ* whether grassland plants vary in their ability to compete for, and utilize peptides, which have recently been shown to act as an N source for plants in strongly N-limited ecosystems. We hypothesized that plants would preferentially use NO_3_^−^-N and NH_4_^+^-N over dissolved organic N in high-productivity grassland where inorganic N availability is high. On the other hand, in low-productivity grasslands, where the availability of dissolved inorganic N is low, and soil availability of dissolved organic N is greater, we predicted that plants would preferentially use N from amino acids and peptides, prior to microbial mineralization. Turves from two well-characterized grasslands of contrasting productivity and soil N availability were injected, in situ, with mixtures of ^15^N-labeled inorganic N (NO_3_^−^ and NH_4_^+^) and ^13^C^15^N labeled amino acid (l-alanine) and peptide (l-tri-alanine). In order to measure rapid assimilation of these N forms by soil microbes and plants, the uptake of these substrates was traced within 2.5 hours into the shoots of the most abundant plant species, as well as roots and the soil microbial biomass. We found that, contrary to our hypothesis, the majority of plant species across both grasslands took up most N in the form of NH_4_^+^, suggesting that inorganic N is their predominant N source. However, we did find that organic N was a source of N which could be utilized by plant species at both sites, and in the low-productivity grassland, plants were able to capture some tri-alanine-N directly. Although our findings did not support the hypothesis that differences in the availability of inorganic and organic N facilitate resource partitioning in grassland, they do support the emerging view that peptides represent a significant, but until now neglected, component of the terrestrial N cycle.

## Introduction

Recent observations of direct amino acid and peptide utilization by plants have challenged the traditional view of soil nitrogen (N) cycling, whereby plants depend entirely on inorganic nitrogen to meet their N demands (Näsholm et al. 1998; Komarova et al. 2008; Hill et al. 2011a,b; Soper et al. 2011). Evidence of intact plant uptake of organic N has been obtained mainly from N-limited ecosystems (e.g., Chapin et al. 1993; Näsholm et al. 1998; Nordin et al. 2001; Henry and Jefferies 2003; Hill et al. 2011a), where soil microbial activity and N mineralization rates are low, and annual plant requirements for N can be 2- to 6-fold greater than the annual inorganic N supply (Giblin et al. 1991; Fisk and Schmidt 1995; Kielland 2001). There is also evidence, however, that plants of more productive ecosystems, including agricultural plants, are able to take up organic N intact (e.g., Streeter et al. 2000; Bardgett et al. 2003; Harrison et al. 2007; Hill et al. 2011b), although inorganic N often appears to be the primary source of N for most plants in ecosystems where N is not limiting.

The ability of plants to compete directly with soil microbes for N has been widely debated; until recently, it was assumed that plants use N that has been mineralized, and only when in excess of microbial demand (Runge 1971). However, Schimel and Bennett (2004) proposed that the form of the captured N depends on the N-limitation of the site. In very N-limited ecosystems, mineralization is minimal and plants and microbes compete for N at the organic monomer stage, with plants capturing amino acid-N as it diffuses from N-rich to N-poor soil microsites. As ecosystems become less N-limited, and mineralization of solid organic N increases, the dynamics of plant–microbial competition also shift, with plants capturing mineralized-NH_4_^+^ as it diffuses through the soil. Finally, in more fertile ecosystems, plants are thought to be poor competitors for organic N, and indeed have no need to compete with microbes, as NH_4_^+^ and NO_3_^−^ are available in excess of microbial demand (Hodge et al. 1998, 1999; Owen and Jones 2001; Jones et al. 2004). However, evidence from both glasshouse (Näsholm et al. 2000; Weigelt et al. 2005) and field (Streeter et al. 2000; Bardgett et al. 2003; Harrison et al. 2007) experiments demonstrate that many grassland species, from both high-productivity grasslands, to extensive, low-productivity sites, are able to take up amino acids directly. As a result of this uncertainty, there remains a need to test how shifting dominance of dissolved N forms influences plant–microbial competition across grasslands of varying productivity and land management intensity.

It has been proposed that in situations where organic N use by plant species is common, there is potential for niche partitioning between species based on the chemical form of N to facilitate species coexistence and maintain plant diversity (McKane et al. 2002). There is evidence from a number of studies to support this theory, including results of laboratory studies showing that plant species from strongly N-limited alpine ecosystems differ in their ability to utilize different chemical forms of N, which points to plant species having fundamental niches based on N form in these situations (Miller and Bowman 2002, 2003). Also, in arctic tundra, dominant plant species were found to utilize the most available N forms *in situ* (i.e., glycine and ammonium), whereas subordinate species mainly used nitrate (McKane et al. 2002). Moreover, in the maritime Antarctic, where peptidic-N is a large component of the soil N pool, Antarctic Hairgrass (*Deschampia Antarctica* Desv.) was found to have higher shoot recovery of small-chain peptide-^15^N over inorganic ^15^N following ^15^N-labeled substrate addition, unlike its competitor, the moss *Sanoinia uncinata* (Hedw.) Loeske, which had higher recovery of NH_4_^+^–^15^N in its shoot material (Hill et al. 2011a). Interspecies differences in N uptake patterns have also been found in grassland species in monoculture, suggesting that they too have fundamental niches based on chemical N form (Weigelt et al. 2005; Harrison et al. 2008). However, it is unclear whether such niches are realized in nature, given that coexisting grassland plants have been shown to principally utilize inorganic N over amino acid-N over longtime periods (2–33 days) (Harrison et al. 2007). However, a variety of fast- and slow-growing grassland plant species have been shown to be differentiated in their uptake of inorganic and organic N in pot experiments (Weigelt et al. 2005; Harrison et al. 2008), and coexisting grassland plant species have been found *in situ* to have complementary N-use strategies based on spatial, temporal, and chemical (NO_3_^−^ vs. NH_4_^+^) pools (Kahmen et al. 2006). Therefore, further direct comparisons of organic N (including peptide) uptake and partitioning between species *in situ* are required to assess the extent of niche differentiation, if any, between grasslands of varying productivities and N availability.

**Figure 1:**
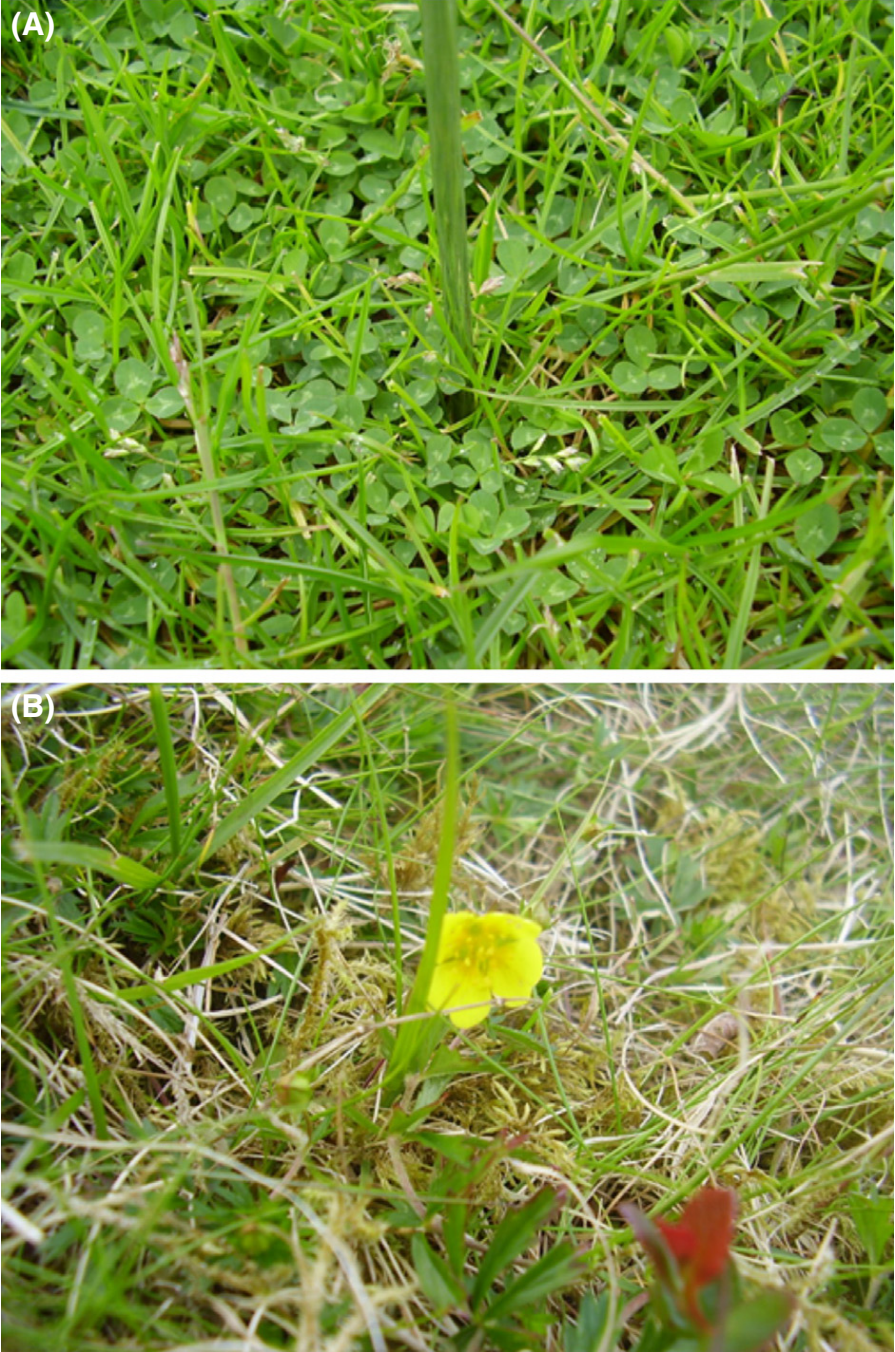
A typical turf harvested following ^15^N-labeled IN and ^15^N^13^C-labeled ON addition, from (A) the high-productivity grassland and (B) the low-productivity grassland.

Here, we tested whether the capacity of grassland plant communities to: (1) capture and (2) partition the soil N pool based on chemical form is related to the shifting dominance of N forms, which is driven primarily by the rate of dissolved organic N turnover by the microbial community. This was done using *in situ* dual-labeling (^15^N^13^C) of turves within two grasslands of contrasting productivity and N availability located along a well-characterized grassland productivity gradient in North Wales, United Kingdom (Fig.1, Table1; Farrell et al. 2011a). We used ^15^N–NH_4_^+^ and ^15^N–NO_3_^−^, as well as dual labeled (^15^N^13^C) amino acids and peptides; the uptake of the latter has not previously been recorded in temperate grasslands, despite dissolved organic N pools in grasslands typically comprising of more peptidic-N than free amino acid-N (Farrell et al. 2011a,b). Specifically, we tested the following hypotheses: (1) Plant species will be more likely to compete directly with microbes for organic N forms, including peptide, in the low-productivity grassland where dissolved ON concentrations are higher and plant available N is more limited (i.e., lower concentrations of NO_3_^−^ and NH_4_^+^); and (2) this will lead to a greater degree of niche partitioning, or utilization of a broader range of chemical N forms by different plant species, than that of the high-productivity grassland plant community. As a secondary objective, we aimed to establish, for the first time in temperate grasslands, the extent to which a range of coexisting plant species of contrasting productivity are able to short circuit the traditional N cycle by directly utilizing peptidic-N *in situ*.

**Table 1:** Characteristics of the 2 grassland sites (adapted from Farrell et al. 2011a,b)

	High-productivity grassland	Low-productivity grassland
Location (WGS84 Lat/Lon)	53°14′11.25″N, 004°01′08.23″W	53°13′29.48″N, 004°01′50.08″W
Altitude (m)	15	320
Above-ground net primary productivity (g DW m^−2^ day^−1^)	5.39 ± 0.36^*^	0.92 ± 0.34^*^
Above-ground C (g C m^−2^)	95.2 ± 4.7	65.1 ± 4.9
Above-ground N (g N m^−2^)	6.60 ± 0.66^*^	2.24 ± 0.17^*^
Plant diversity (Shannon-*H*)	0.64 ± 0.09^*^	2.22 ± 0.05^*^
Soil moisture content (%)	29.1 ± 1.0^*^	44.3 ± 2.4^*^
Soil organic matter (%)	7.8 ± 0.5^*^	22.3 ± 2.0^*^
Bulk density (g cm^−3^)	1.09 ± 0.01^*^	0.45 ± 0.08^*^
pH	6.92 ± 0.28^*^	4.76 ± 0.04^*^
Electrical conductivity (*μ*S cm^−1^)	103 ± 27	36 ± 4
Total soil N (g N m^−2^)	562 ± 55	646 ± 104
DON (mg N m^−2^)	169 ± 58	301 ± 74
NH_4_^+^–N (mg N m^−2^)	132 ± 26	73.4 ± 36.8
NO_3_^−^–N (mg N m^−2^)	401 ± 50^*^	0.6 ± 0.5^*^
Free amino acid-N (mg N m^−2^)	0.54 ± 0.18	0.28 ± 0.11
Peptide-N (mg N m^−2^)	1.74 ± 0.27	1.71 ± 0.64

Significant differences (*P *<* *0.05) between sites are indicated by an asterisk. Soil data are presented on a dry mass basis by area to a depth of 15 cm.

## Materials and Methods

### Site description

Stable isotope labeling was carried out *in situ* at two contrasting sites situated along a well-characterized grassland productivity gradient at Abergwyngregyn, Gwynedd, Wales, UK (53°14 11.25 N, 004°01 08.23 W, and 53°13 29.48 N, 004°01 50.08 W, respectively) (Farrell et al. 2011a). The first site (site 1 of Farrell et al. 2011a; hereafter referred to as “high-productivity grassland”) was a highly productive (i.e., high aboveground biomass productivity), inorganic N-(in particular NO_3_^−^) rich Eutric Cambisol soil (DIN:DON = 3.15), that had received regular applications of inorganic fertilizer (120 kg N ha^−1 ^year^−1^), and was dominated by the grass *Poa trivialis* L. and white clover (*Trifolium repens* L.) (Fig.1A). The second site (site 3 of Farrell et al. 2011a; hereafter referred to as “low-productivity grassland”) was an unfertilized, lower productivity, but more species-rich, *Agrostis-Festuca* upland grassland on an organic matter-rich Cambic Podzol (Fig.1B), with a dissolved N pool rich in organic N (DIN:DON = 0.25; based on the findings of Farrell et al. 2011a). Further site details are given in Table1, and in Farrell et al. (2011a).

### Experimental design and sampling

At each grassland site, 5 replicate blocks, each containing 5, 20 × 20 cm turves, were marked out approximately 2 weeks prior to the labeling event over an area of c. 0.25 ha. In June 2011, randomly allocated solutions containing a range of N forms were injected into the root zone of each turf simultaneously at each site. These solutions contained a mixture of four N forms: potassium nitrate (KNO_3_), ammonium sulfate ((NH_4_)^2^SO_4_), the amino acid alanine, and the peptide tri-alanine. We chose alanine as the amino acid monomer as it occurs commonly as free amino acids and short peptides in both grassland sites (Farrell et al. 2011a), and it has been found to be taken up by plant roots in both sterile conditions (Hill et al. 2011b) and *in situ* by an Antarctic grass (Hill et al. 2011a).

The solutions were made up of equal concentrations of the individual N forms (1.4 mg N turf^−1^, 100 *μ*mol N turf^−1^) in 50 mL double-distilled water and were injected into the soil (0–8 cm depth) evenly across the turf in 10 × 5 mL injections. The amount of N added to the turves needed to be sufficient enough to allow for detection of ^15^N and ^13^C within the plant and microbial biomass; however, we are aware that this may have produced enrichment and dilution effect for the certain N forms at the two sites (total N added equivalent to 35 mg N m^−2^, and 8.8 mg N m^−2^ per N form added). All turves received the same mixture of N forms, but within each block, individual turves were allocated randomly assigned treatments in which only one of each of the four N forms was isotopically labeled with ^15^N (25 *μ*mol ^15^N turf^−1^) and ^13^C for the amino acid (alanine, U–^13^C^3^, 97–99%; ^15^N, 97–99%) and peptide (tri-alanine, U–^13^C^3^, 97–99%; ^15^N, 97–99%; CK Gas Products, Hook, UK). This allowed us to test the ability of plant and microbes to utilize N from the labeled substrates, and it also enabled us to test for direct uptake of the amino acid and peptide by plants, as indicated by plant tissue enrichment of both ^13^C and ^15^N (Näsholm et al. 1998). A fifth set of turves within each block were injected with distilled H_2_O as a natural abundance control.

Within each turf, solutions (50 mL) were injected into the top 8 cm of soil in 10 consecutive injections across a grid template using the side-port needle technique described by Streeter et al. (2000). Previous field studies have used chase periods spanning days or even weeks (e.g., Bardgett et al. 2003; Harrison et al. 2007). However, when comparing inorganic and organic N uptake, short-term labeling is a more effective way of assessing plant–microbe competition events, as the residence time of organic N within soil is of the order of a minutes due to rapid microbial uptake, whereas forms of N less desirable to soil microbes, such as nitrate, are often taken up much more slowly (Jones et al. 2005b; Hill et al. 2012; Wilkinson et al. 2014). Therefore, we destructively harvested turves to a depth of 10 cm after a much shorter time period (2.5 h). This provides sufficient time to harvest turves once labeled_N has been injected, yet reduces the plant uptake of inorganic-^15^N following microbial mineralization and turnover of organic-^15^N. The bulk of inorganic-^15^N is likely released during a slow secondary mineralization phase, given that half times for organic N mineralization in these two grasslands range from 6–14 h (Wilkinson et al. 2014). A number of subsamples of shoot material (approximately 1–3 g total dwt) of coexisting species that were consistently present in each quadrat (site 1: *Poa trivialis* L. and *Trifolium repens* L.; site 2: *Agrostis capillaris* L., *Anthoxanthum odoratum* L., *Festuca ovina* L., *Luzula* sp. and *Potentilla erecta* (L.) Raeusch.) were taken across each turf, and subsamples of the same species were bulked together to give one sample of each species present for ^13^C/^15^N analysis. Furthermore, three subsamples of root and soil material were taken from across each turf using a 1-cm-diameter hand corer, and these were also bulked together to produce a soil sample and a root sample from each turf for ^13^C/^15^N analysis. Finally, the remaining turf was cutout of the ground to a depth of 8 cm. Soil for ^13^C/^15^N analysis was passed through a 3-mm sieve, and the chloroform fumigation-extraction procedure (Vance et al. 1987) was immediately carried out in the laboratory, at the nearby Henfaes field station (Bangor University) in order to determine uptake of ^15^N and ^13^C by the soil microbial biomass. K_2_SO_4_ extractions were carried out on fumigated and non-fumigated soil, and these were freeze-dried and a subsample was analyzed for C and N and ^13^C and ^15^N content at the NERC Life Sciences Mass Spectrometer Facility, Centre for Ecology and Hydrology, Lancaster (precision for working standards better than 0.46 ‰ (^13^C) and 6.92 ‰ (^15^N)). Samples (1 mg) were combusted in a Carlo Erba elemental analyzer, and the resultant CO_2_/N_2_ from combustion and reduction analyzed for *δ*^13^C/^15^N using an isotope ratio mass spectrometer (IRMS; Dennis Leigh Technologies).

Plant shoot and root material for ^13^C/^15^N analysis were immediately frozen following the harvest and transported from the study sites to the laboratory at Lancaster. Here, roots were washed in 0.5 mol/L CaCl_2_ solution to remove any of the isotope label attached externally, and plant material was dried for 48 h at 70°C before being weighed, ground and analyzed for C and N and ^13^C and ^15^N content at the NERC Life Sciences Mass Spectrometer Facility, as described above.

The remaining intact turves were separated completely into the shoots of the most abundant species at each site, roots and soil. These components were also dried for 48 h at 70°C and weighed to obtain a measure of total turf and turf component biomass.

### Data analysis

Values of *δ*^13^C/^15^N were converted into atom % values using the equations: 

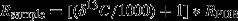
1where R is the ratio of ^13^C to ^12^C and R_PDB_ is the natural abundance standard for C or N. 

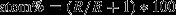
2

Atom % excess values were calculated by subtracting control atom % values from treatment atom % values:

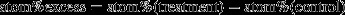
3

For ^15^N enrichment calculations, control treatments consisted of the plots injected with dH_2_O. For ^13^C calculations, control treatments consisted of plots injected with dH_2_O, and inorganic (NO_3_^−^ and NH_4_^+^) N treatments. Due to high levels of variation in natural abundance levels of ^13^C, exacerbated by the very large C pool size relative to injected quantities of C, the lowest natural abundance atom % value was used in equation 3 to avoid negative values of 13C enrichment.

Atom % excess values in the plant and microbial biomass were converted into total sample concentration values (nmol ^15^N/^13^C excess g^−1^ plant/microbial biomass) using the following equations: 

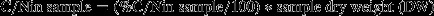
4

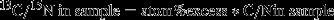
5

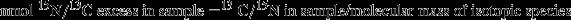
6

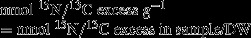
7

We used analysis of variance (ANOVA) followed by post hoc Tukey tests to assess for differences between sites in the recovery of ^15^N and ^13^C from the added N forms within microbial biomass and plant material. We also performed ANOVA's to determine which of the N forms were recovered in the greatest amounts in both the microbial biomass and in the plant tissue, as well to assess plant and microbial competition for the different N forms. All analyses were carried out on recovered ^15^N/^13^C data (as a percentage of the total amount of isotope added), with site (high- vs. low-productivity grassland), turf fraction (microbial biomass vs. root/shoot biomass), N form (NO_3_^−^, NH_4_^+^, alanine, and tri-alanine) and block included in the analysis as factors. Where necessary, data were transformed in order to meet the assumptions of normality and homogeneity of variance. Where significant differences in the recovery of ^15^N or ^13^C occurred between sites, or between microbial biomass and plant material, we also performed ANOVA's on tissue isotope concentrations (nmol ^15^N/^13^C excess g-^1^ DW) in order to assess whether differences between sites or between microbial biomass and plant material were driven by biomass differences between sites or between plant material and microbial biomass.

To assess the partitioning of N forms between different plant species, we carried out ANOVA's followed by Tukey tests on data from each site separately. Within each site, analyses were carried out on ^13^C and ^15^N concentration data (nmol ^13^C/^15^N excess g^−1^ DW) for the shoot material of each species, with N form (NO_3_^−^, NH_4_^+^, alanine, and tri-alanine) and block included in the analysis as factors. Again, where data did not meet the assumptions of normality and homogeneity of variances, analyses were performed on transformed data.

Regression analyses were performed to identify linear relationships between concentrations (nmol excess g^−1^ DW) of ^13^C and ^15^N in microbial biomass, roots and the shoot material of each species at both sites. All analyses were performed using IBM SPSS Statistics (Version 20.0. Armonk, NY: IBM Corp).

## Results

### Differences in total recovery of substrate in plant and microbial biomass between grasslands

**Figure 2:**
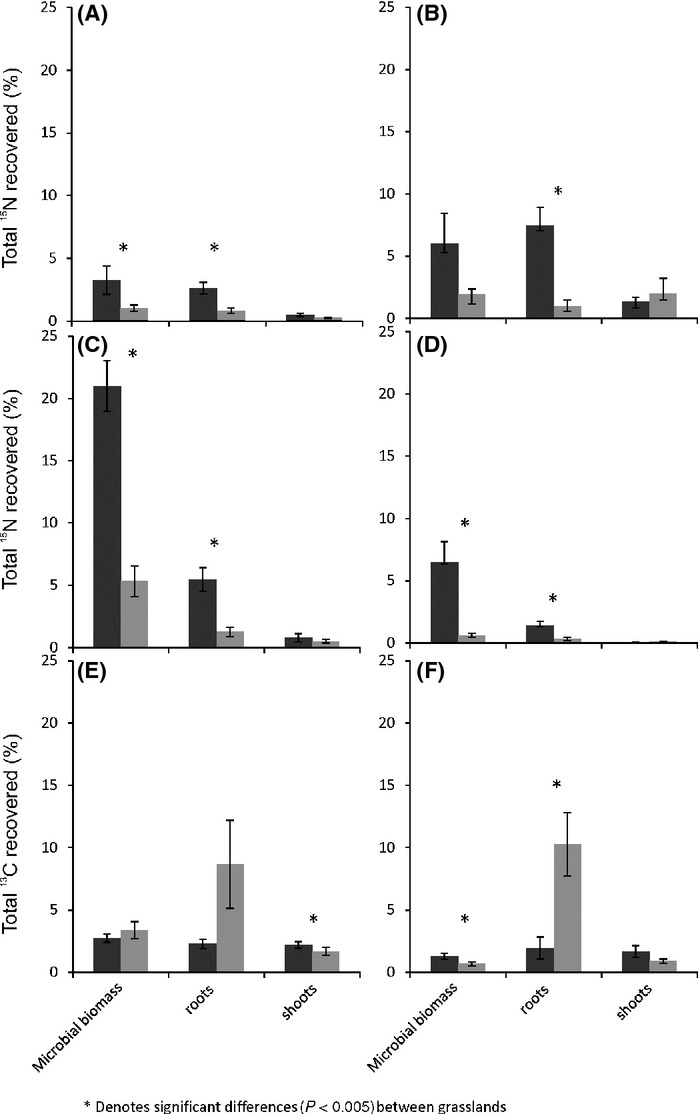
The total percentage of added isotope recovered from within the total microbial, root and shoot biomass, following the ^15^N-labeled application of (A) NO_3_^−^, (B) NH_4_^+^, (C) alanine, and (D) tri-alanine, and the ^13^C-labeled application of (E) alanine, and (F) tri-alanine, in the high-productivity (black bars) and low-productivity (gray bars) grasslands.

In both grasslands, we only recovered a small percentage of the ^15^N and ^13^C that was originally added after 2.5 h, and this ranged from 0.4–26.1% and 0–9.1% of added ^15^N, and 0.36–5.3% and 0.25–18.4% of added ^13^C in the high- and low-productivity grasslands respectively (Fig.2). There were significant differences between grasslands in the recovery of ^15^N from all substrates (Fig.1A–D; *F*_(1,169) _= 33.86, *P* < 0.001), with a higher percentage of ^15^N recovered in plant and microbial biomass in the higher productivity grassland. For example, microbes took up significantly more alanine-^15^N (*F*_(1,3) _= 118.20, *P* = 0.002) and tri-alanine-^15^N (*F*_(1,4) _= 103.02, *P* = 0.001), and marginally more NO_3_-^15^N (*F*_(1,3) _= 10.537, *P* = 0.048) in the high-productivity grassland compared to the low-productivity grassland. Within the microbial biomass, organic-^15^N was recovered in higher quantities in the high-productivity grassland, regardless of biomass differences between sites (Fig S1A,B), with concentrations of microbial ^15^N (^15^N excess g^−1^ soil DW) being 1.8x greater in the high-productivity grassland following alanine addition (*F*_(1,3) _= 13.88, *P* = 0.034), and 4x greater following tri-alanine addition (*F*_(1,4) _= 72.45, *P* = 0.001; Fig. S2A).

There were no significant differences between grasslands in the recovery of ^15^N within shoot material following any of the labeled substrate additions. However, significantly more ^15^N was recovered in the root material from the high-productivity grassland compared to the low-productivity grassland following the addition of all labeled substrates (Fig.2A–D; ^15^N–NO_3_: *F*_(1,4) _= 22.36, *P* = 0.009, ^15^N–NH_4_: *F*_(1,3) _= 19.93, *P* = 0.021, ^15^N^13^C-alanine: *F*_(1,3) _= 42.57, *P* = 0.007, and ^15^N^13^C-tri-alanine: *F*_(1,4) _= 50.36, *P* = 0.002). These differences between grasslands were also significant when differences in root biomass between sites were taken into account: Root biomass was much greater in the low-productivity grassland (Fig. S1C), yet concentrations of ^15^N (^15^N excess g^−1^ DW) within root biomass in the high-productivity grassland were between 8–15 times greater than in the low-productivity grassland (Fig. S2B; *P* < 0.011).

There were also some differences between grasslands in ^13^C recovery within plant material following the addition of labeled organic N forms (Fig.2E and F), although this did not follow any discernible pattern. For example, significantly more ^13^C was recovered in shoot material following labeled alanine addition (*F*_(1,4) _= 13.71, *P* = 0.021) in the high-productivity grassland compared to the low-productivity grassland. However, significantly less ^13^C was recovered in the root material following labeled tri-alanine addition in the high-productivity grassland (*F*_(1,4) _= 41.40, *P* = 0.003). These differences are probably related to differences in root and shoot biomass between sites as no significant differences were observed in root and shoot tissue concentrations of ^13^C between grasslands.

### Contrasting patterns of recovery of inorganic and organic N within plant and microbial biomass

**Figure 3:**
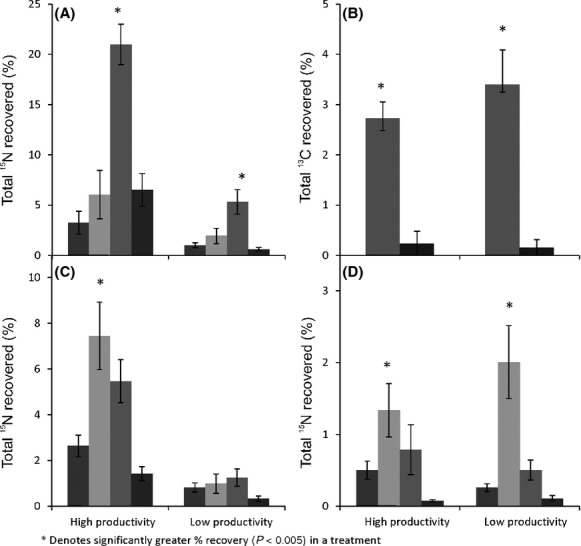
The total percentage of added (A) ^15^N recovered from within microbial biomass, (B) ^13^C recovered from within microbial biomass, (C) ^15^N recovered from within root material, and (D) ^15^N recovered from within shoot material, following the ^15^N-labeled application of NO_3_^−^ (black bars), NH_4_^+^ (pale gray bars), alanine (dark gray bars), and tri-alanine (white bars) in the high-productivity and low-productivity grasslands.

In both grasslands, more ^15^N was recovered in the microbial biomass following labeled alanine addition (Fig.3A; high-productivity grassland: *F*_(3,10) _= 13.17, *P* = 0.001, low-productivity grassland: *F*_(3,12) _= 7.72, *P* = 0.004), when compared to other forms of N addition (although this was not significantly greater than ^15^N–NH_4_ in the low-productivity grassland). Likewise, ^13^C recovery within the microbial biomass was greater following alanine addition than with tri-alanine addition in both grasslands (Fig.3B; high-productivity grassland: *F*_(1,3) _= 15.31, *P* = 0.03, low-productivity grassland: *F*_(1,4) _= 33.51, *P* = 0.004). Within root material, more ^15^N was recovered in the ^15^N–NH_4_ treatment in the high-productivity grassland (Fig.3C; *F*_(3,12) _= 10.21, *P* = 0.001), although this was not significantly greater than the amount recovered under the labeled alanine treatment. Recovery of ^15^N within shoot material was higher following ^15^N–NH_4_ addition in both grasslands (Fig.3D), although this was only significantly greater than the recovery of ^15^N from tri-alanine in the high-productivity grassland (*F*_(3,12) _= 13.03, *P* > 0.001) and the recovery of ^15^N from NO_3_^−^ and tri-alanine in the low-productivity grassland (*F*_(3,12) _= 13.51, *P* > 0.001). No differences in root recovery of ^15^N were observed between treatments in the low-productivity grassland, nor were any significant differences observed in either grassland between the recovery of ^13^C following labeled amino acid and peptide addition in all plant material.

### Plant and microbial competition for different N forms

**Figure 4:**
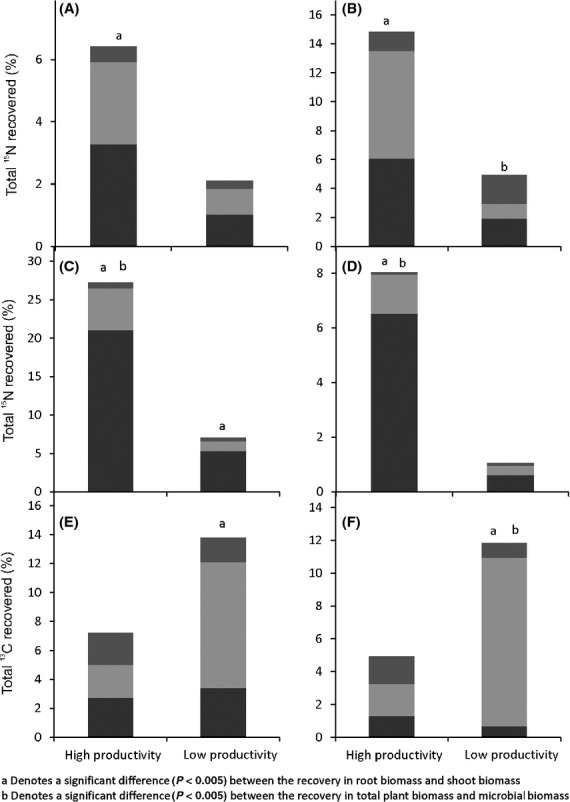
The total percentage of added ^15^N recovered from within the microbial biomass (black bars), root material (pale gray bars), and shoot material (dark gray bars), following the ^15^N-labeled application (A) NO_3_^−^, (B) NH_4_^+^, (C) alanine, and (D) tri-alanine, and ^13^C following the ^13^C-labeled application (E) alanine and (F) tri-alanine.

Generally, there were very few differences between the amount of ^15^N and ^13^C recovered in plant and microbial biomass (Fig.4), although there were some exceptions. In the high-productivity grassland, three times more ^15^N was recovered in the microbial biomass compared to plant biomass following organic N addition (Fig.4C; alanine: *F*_(1,3) _= 23.15, *P* = 0.017, and Fig.4D; tri-alanine: *F*_(1,4) _= 38.30, *P* = 0.003). In the low-productivity grassland, slightly more ^15^N was recovered in plant biomass compared to microbial biomass following labeled NH_4_^+^ addition (Fig.4B; *F*_(1,4) _= 10.22, *P* = 0.033), and seventeen times more ^13^C was recovered in plant biomass (Fig.4F; *F*_(1,4) _= 51.85, *P* = 0.002) following labeled tri-alanine addition. Nonetheless, in most cases, equal amounts of ^13^C and ^15^N from added substrates were recovered in plant material and microbial biomass in both grasslands. However, plant material always had a much higher concentration of ^15^N and ^13^C (nmol excess g^−1^) than microbial biomass in all substrate addition treatments (Fig. S3).

In the high-productivity grassland, recovery data from within plant material indicate that added ^15^N was largely retained in root material (between 1.4–7.5% of added N), with significantly smaller amounts (between 0.5% and 1.3%) recovered in shoot biomass following the addition of labeled NO_3_^−^ (*F*_(2,7) _= 9.67, *P* = 0.01), NH_4_^+^ (*F*_(2,8) _= 5.46, *P* = 0.032), alanine (*F*_(2,7) _= 32.15, *P* < 0.001) and tri-alanine (*F*_(2,8) _= 95.70, *P* < 0.001). However, in the low-productivity grassland, ^15^N was recovered in equal amounts from root and shoot material, with the exception of alanine addition, where again most ^15^N was retained in the root material (*F*_(2,7)_=39.86, *P* < 0.001). Furthermore, most ^13^C was retained in the root material (Fig.4E; alanine: *F*_(2,7) _= 7.30, *P* = 0.019, tri-alanine: *F*_(2,8) _= 44.22, *P* < 0.001) in the low-productivity grassland, whereas there were no differences in the recovery of ^13^C between root and shoot material in the high-productivity grassland.

### Plant species-specific differences in substrate ^15^N and ^13^C concentrations

**Figure 5:**
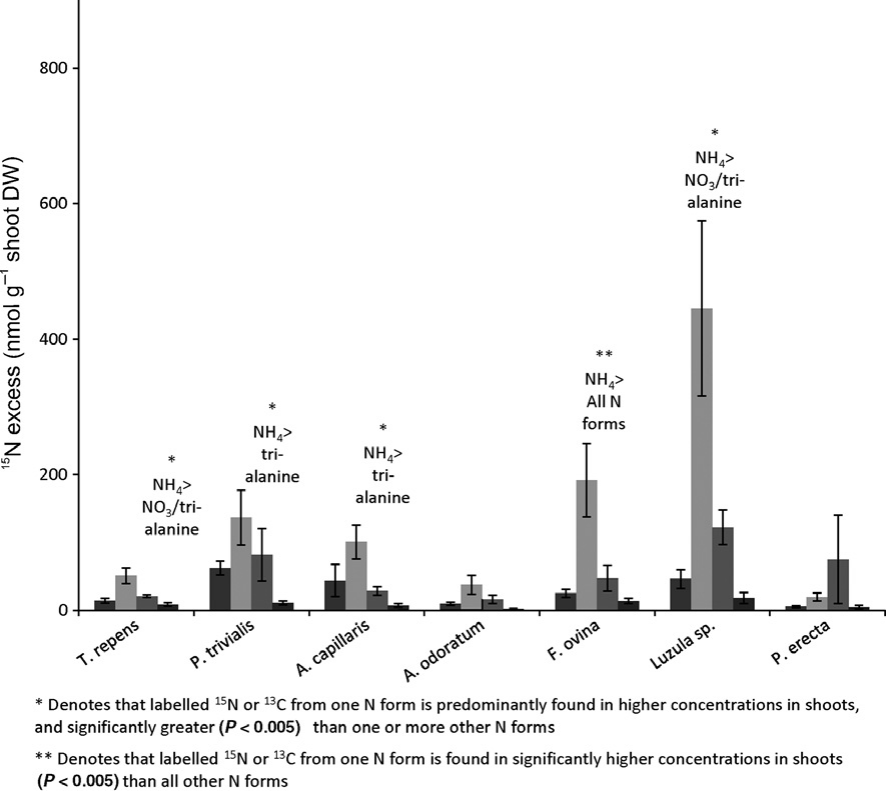
Concentrations of ^15^N in the shoot material of species in the high-productivity grassland (*T. repens* and *P. trivialis*) and the low-productivity grassland (*A. capillaris*, *A. odoratum*, *F. ovina*, *Luzula* sp., and *P. erecta*), following the application of isotopically labeled ^15^N–NO_3_^−^ (black bars), ^15^N–NH_4_^+^ (pale gray bars), and ^13^C^15^N-alanine (dark gray bars) or ^13^C^15^N-tri-alanine (white bars).

We found no clear evidence for resource partitioning between coexisting species in either the high- or the low-productivity grassland. In both grasslands, the highest shoot ^15^N concentrations for most species were found following the addition of NH_4_–^15^N, although in most cases, this was not significantly different to either NO_3_–^15^N or alanine–^15^N (Fig.5). Furthermore, in the low-productivity grassland, no significant differences were observed between ^15^N shoot concentrations of either *A. odoratum* or *P. erecta* following any of the labeled substrate additions.

In both grasslands, we found no significant differences in ^13^C enrichment of shoots of coexisting plant species between alanine and tri-alanine treatments, with the exception of *Luzula* sp. in the low-productivity grassland, in which 340% more alanine-^13^C (540 nmol ^13^C g^−1^ DW increase) was measured in shoot tissue than tri-alanine-^13^C (*F*_(1,3) _= 18.13, *P* = 0.024).

### Evidence of direct plant uptake of ON

We detected significant, but weak, relationships between the excess concentrations of tri-alanine-^13^C and ^15^N in both root material (*R*^2^ = 0.824, *P* = 0.033) and the shoot material of *Luzula* sp. (*R*^2^ = 0.795, *P* = 0.042) in the low-productivity grassland (Fig. S4). In the high-productivity grassland, however, we only found a significant regression between the isotope concentrations in the microbial biomass following labeled alanine addition (*R*^2^ = 0.955, *P* = 0.023), and not in plant material.

## Discussion

Our findings illustrate that plants in grasslands are able to acquire N from both inorganic and organic sources (including peptides); however, regardless of grassland productivity and availability of dissolved inorganic and organic N in soil, most plant N was taken up as inorganic N, and particularly NH_4_ ^+^ . This supports the traditional role of plants predominately using inorganic N and refutes our hypothesis that patterns of plant N uptake change with shifts in the availability of dissolved inorganic and organic N, thus allowing plant species to coexist through using distinct pools of dissolved N. However, the simultaneous uptake of tri-alanine-^13^C by all plant species tested, coupled with significant positive relationships between tri-alanine-^13^C and ^15^N concentrations in root material and *Luzula* shoots in the low-productivity grassland, suggests that a proportion of peptide-N was taken up intact by the plant communities within both grasslands (Näsholm et al. 1998, 2000; Nordin et al. 2001). Thus, we were able to show, for the first time in both low- and high-productivity temperate grassland, that plants are able to assimilate N from peptides *in situ*.

Our data do not support the notion that plant utilization of dissolved organic N is more prevalent as inorganic N becomes less available within the soil-dissolved fraction. On the one hand, uptake of inorganic N by plant roots was greater in the high-productivity grassland, which suggests that plants of these grasslands are adapted to utilizing the most abundant forms of N. But on the other hand, more NH_4_^+^ was taken up by plants compared to NO_3_^−^, which was the most abundant N form in the high-productivity soil. In addition, we found that uptake of organic N in the form of amino acids and peptides by plant roots was greater in the more productive grassland, even when differences in root biomass between sites were considered. This highlights the fact that soil solution concentrations of N forms may be poor indicators of their flux into plants or soil microbes.

Harrison et al. (2007) showed that coexisting plant species of semi-natural, low-productivity grassland utilize a range of N forms, but all take up larger quantities of inorganic N over organic N, and simple amino acids over more complex amino acids. We also found that most plants from both high- and low-productivity grasslands had higher shoot concentrations of ^15^N in the ^15^NH_4_^+^ treatment (with the exception of *Potentilla erecta*). In most cases, these ^15^N concentrations were not significantly different to those in the NO_3_^−^ or alanine treatments, which were also relatively high. We examined how differences in the isotope dilution within soil N pools affect plant uptake of ^15^N by scaling ^15^N excess values recorded within shoot tissue of coexisting species based on the size of the target N pool within each grassland. We found that 50–500x more ^15^N–NH_4_ was recovered in shoot material than dissolved ON forms across all species, although these calculations do not consider the flux of dissolved ON through the soil pool, which can be extremely rapid (e.g., Farrell et al. 2011b; Hill et al. 2012; Wilkinson et al. 2014). This indicates that, even when pool dilution is considered, coexisting plant species in high- and low-productivity grasslands principally utilize N from inorganic sources over organic N forms, regardless of soil-dissolved IN/ON concentrations.

Schimel and Bennett (2004) proposed that in strongly N-limited environments where dissolved organic N dominates the soil profile, plants are able to capture organic N as it diffuses through the soil. As N availability increases, more organic N is mineralized and plants and microbes actively compete for NH_4_^+^. Although soil concentrations of NO_3_^−^ were much higher in the high- than low-productivity grassland (Table1), we found no significant differences in concentrations of soil NH_4_–N or total soil N. However, NO_3_–N occurs in high concentrations in the high-productivity grassland (over 3x the amount of NH_4_^+^–N, and 240–750 ×  more than dissolved ON forms; Table1; Farrell et al. 2011a,b). When pool dilution is considered, nitrate uptake in the shoot material of the two species in this grassland ranges between 10–46 *μ*mol ^15^N excess g^−1^, which is much greater than that of the dissolved ON forms, which range from 11.5–81.8 nmol ^15^N excess g^−1^. Nevertheless, recovery of ^15^N from NH_4_ is still 20% higher than that from NO_3_ in *T. repens,* although in *P. trivialis*, recovery of ^15^N from NH_4_ is 28% lower than from NO_3_. When pool dilution is considered, therefore, our data still show that coexisting species take up more inorganic N than organic N, however, in the low-productivity, inorganic N-rich grassland, niche differentiation between species may potentially occur based on inorganic N form. However, this would need to be confirmed by further experimentation that considers inorganic N fluxes alongside pool dilution.

It is also important to note that enrichment of shoot material is not necessarily indicative of total plant uptake of different compounds. Translocation of N from roots to shoots can occur at different rates depending on chemical form; inorganic N can be translocated to shoot material faster than organic N in grassland plants, despite the equally high amounts of the different N forms sequestered in the root material (Weigelt et al. 2005). Consequently, it is possible that N enrichment in the shoot material in our study may under- or over-estimate the preferences of certain species for more complex organic N forms. As we did not measure root-N concentrations at the species level, it is not possible to say whether certain species were retaining larger concentrations of organic N belowground.

Plant utilization of amino acids and peptide-N occurred across both grassland types. In both grasslands, plants were very strong competitors with microbes for all forms of N. It may be that at both sites, organic N, and in particular alanine, was rapidly mineralized and plants were taking up high levels of ^15^N as mineralized NH_4_ ^+^ . This could be occurring more in the high-productivity grassland, where more organic-^15^N was recovered in plant roots compared to the low-productivity grassland, but less organic-^13^C. Our data show that while plants and microbes utilize N from both inorganic and organic sources, more organic N, and particularly alanine-N, was recovered from within microbial biomass, while more NH_4_–N was recovered from within plant material, when compared to other forms of N. Previous laboratory studies using soil from both the high- and low-productivity grasslands have demonstrated that amino acids and peptides are removed from soil solution by microbes in a matter of minutes and that the C is mineralized rapidly (substrates have halftimes of between 5–7 minutes for alanine and 6–9 minutes for tri-alanine, with faster uptake and mineralization rates occurring in the higher productivity soil; Wilkinson et al. 2014). This suggests that much of the added organic N would probably become unavailable for plant uptake very quickly and that rapid mineralization of the substrates could account for a significant proportion of organic ^15^N recovered in the plant material in both grasslands.

Values of *δ*^13^C obtained from plant and microbial biomass samples fluctuated greatly. However, within both grasslands, plant material was enriched in ^13^C, which implies that plants were, to some degree, taking in alanine and tri-alanine intact. In particular, a high percentage of ^13^C from tri-alanine was recovered in plant root material in the low-productivity site, and this was significantly greater than that recovered from within the microbial biomass. Coupled with significant, but weak regressions between isotopes measured in root and shoot material of *Luzula* sp., this indicates a proportion of tri-alanine was probably taken up intact by plants. We accept that using colocation of ^13^C and ^15^N to infer direct uptake is not unequivocal (Näsholm and Persson 2001; Jones et al. 2005a; von Felten et al. 2008; Rasmussen et al. 2010). We therefore remain cautious in drawing conclusions around the amount of organic N taken up directly by *Luzula* sp., or by the roots of the other plant species in the low-productivity grassland. However, it is clear from the partitioning of alanine and tri-alanine C between microbial uptake and mineralization in these soils that not all of the ^13^C recovered in plants can be accounted for by microbially mineralized ^13^C (Wilkinson et al. 2014). Further chemical analysis of plant tissue might be useful in providing absolute confirmation of direct peptide uptake (Bol et al. 2002; von Felten et al. 2008; Harrison et al. 2008), although the amounts of added labelled material were kept as near to field concentrations as possible and therefore precise detection within tissue would probably not be feasible.

A possible mechanism for potential direct peptide uptake by plant species in the low-productivity grassland would be greater interception of organic N as it diffuses from areas of high to low concentration by mycorrhizal fungi (Schimel and Bennett 2004). Moreover, it is possible that some root enrichment values could be attributed to enrichment within microbial symbionts inside the root tissue itself (e.g., arbuscular mycorrhizal fungi). However, the relatively subordinate *Luzula* sp is not noted for being heavily colonized by arbuscular mycorrhizal fungi (Read and Haselwandter 1981; Rose 1989; Grime et al. 2007). An alternative explanation for direct uptake of tri-alanine by *Luzula*, therefore, might be the existence increased peptide transporters within the root system, or the ability to rapidly colonize hot spots of organic N activity within grasslands. These ideas, however, require further testing.

## Conclusions

Our data show that plants in both high- and low-productivity grasslands generally take up larger quantities of inorganic over organic N, thereby providing no support for the idea that plant uptake of inorganic/organic N in temperate grasslands shifts based on the dominant form of dissolved N in soil, as proposed by Schimel and Bennett (2004). Furthermore, we found no clear evidence for resource partitioning of N between coexisting plant species in either high- or low-productivity grasslands. However, plants in both grasslands were found to take up considerable amounts of ^15^N and ^13^C from organic sources, and for the first time, we provide evidence that some direct uptake of organic N in its peptide form by plants occurs in grasslands. We propose that although grassland plant species across gradients of productivity and inorganic N/dissolved organic N availability have the ability to rapidly utilize N from amino acids and peptides, they predominantly utilize inorganic N when available. However, as N availability was still quite high in both grasslands, a more pronounced N availability gradient may unveil shifts in plant organic N uptake and partitioning between species with dissolved organic N availability.

## Supporting Information

**Figure S1.** Differences in (A) microbial C (g C kg^−1^ soil DW), (B) microbial N (g N kg^−1^ soil DW), (C) root DW (g m^2^) and (D) shoot DW (g m^2^) between the high productivity and low productivity grassland. **Figure S2.** Differences in (A) microbial biomass and (B) root tissue concentrations (nmol ^15^N excess g^−1^ DW) of ^15^N between high (black bars) and low (light grey bars) productivity grasslands following labelled-substrate addition. **Figure S3.** Differences in microbial biomass (black bars), root (light grey bars) and shoot (dark grey bars) biomass concentrations of ^15^N (nmol ^15^N excess g^−1^ DW) 2.5 h after the addition of (A) ^15^N-NO_3_, (B) ^15^N-NH_4_, (C) ^15^N^13^C-alanine and (D) ^15^N^13^C tri-alanine, and biomass concentrations of ^13^C (nmol ^13^C excess g^−1^ DW) 2.5 h after the addition of (E) ^15^N^13^C-alanine and (F) ^15^N^13^C tri-alanine. **Figure S4.** Significant regressions of nmol ^13^C excess g^−1^ DW versus nmol ^15^N excess g^−1^ DW for (A) extracts of microbial biomass (black circles) in the high productivity grassland following labelled alanine addition, (B) root material (black circles) in the low productivity grassland following labelled tri-alanine addition, and (C) *Luzula* sp. shoot material (black circles) in the low productivity grassland following labelled tri-alanine addition.
