# Supplementary material for: Challenging the paradigm of nitrogen cycling: no evidence of in situ resource partitioning by coexisting plant species in grasslands of contrasting fertility
Source: Ecol Evol. 2014 Dec 23;5(2):275–87. doi: 10.1002/ece3.1244 (PMC4314261; doi:10.1002/ece3.1244)
Supplement: Supplementary file 1 — Figure S1. Differences in (A) microbial C (g C kg−1 soil DW), (B) microbial N (g N kg−1 soil DW), (C) root DW (g m2) and (D) shoot DW (g m2) between the high productivity and low productivity grassland. Figure S2. Differences in (A) microbial biomass and (B) root tissue concentrations (nmol 15N excess g−1 DW) of 15N between high (black bars) and low (light grey bars) productivity grasslands following labelled-substrate addition. Figure S3. Differences in microbial biomass (black bars), root (light grey bars) and shoot (dark grey bars) biomass concentrations of 15N (nmol 15N excess g−1 DW) 2.5 h after the addition of (A) 15N-NO3, (B) 15N-NH4, (C) 15N13C-alanine and (D) 15N13C tri-alanine, and biomass concentrations of 13C (nmol 13C excess g−1 DW) 2.5 h after the addition of (E) 15N13C-alanine and (F) 15N13C tri-alanine. Figure S4. Significant regressions of nmol 13C excess g−1 DW versus nmol 15N excess g−1 DW for (A) extracts of microbial biomass (black circles) in the high productivity grassland following labelled alanine addition, (B) root material (black circles) in the low productivity grassland following labelled tri-alanine addition, and (C) Luzula sp. shoot material (black circles) in the low productivity grassland following labelled tri-alanine addition. [file ece30005-0275-sd1.docx]

Supplementary Figure 1

*

F(1,46)=86.34,

P<0.001)

*

F(1,48)=565.00,

P<0.001)

*

F(1,46)=54.19,

P<0.001)

*

F(1,46)=83.86,

P<0.001)

Differences in (a) microbial C (g C kg^-1^ soil DW), (b) microbial N (g N kg^-1^ soil DW), (c) root DW (g m^2^) and (d) shoot DW (g m^2^) between the high productivity and low productivity grassland. Significant differences between grasslands are denoted by an asterix (*), and statistical values are presented.

Supplementary Figure 2

*

F_(1,3)_=32.64

P=0.011

*

F_(1,3)_=31.45

P=0.011

*

F_(1,3)_=289.0

P<0.001

*

F_(1,4)_=72.45

P=0.001

*

F_(1,4)_=129.7

P<0.001

*

F_(1,3)_=13.88

P=0.034

Differences in (a) microbial biomass and (b) root tissue concentrations (nmol ^15^N excess g^-1^ DW) of ^15^N between high (black bars) and low (light grey bars) productivity grasslands following labelled-substrate addition. Significant differences between grasslands are denoted by an asterix (*), and statistical values are presented.

Supplementary Figure 3

ᵻ^12^

ᵻ^11^

ᵻ^10^

ᵻ^9^

*^5^

ᵻ^8^

*^4^

ᵻ^7^

*^3^

ᵻ^6^

ᵻ^5^

*^2^

*^1^

ᵻ^4^

ᵻ^3^

ᵻ^2^

ᵻ^1^

Differences in microbial biomass (black bars), root (light grey bars) and shoot (dark grey bars) biomass concentrations of ^15^N (nmol ^15^N excess g^-1^ DW) 2.5 h after the addition of (a) ^15^N-NO_3_, (b) ^15^N-NH_4_, (c) ^15^N^13^C-alanine and (d) ^15^N^13^C tri-alanine, and biomass concentrations of ^13^C (nmol ^13^C excess g^-1^ DW) 2.5 h after the addition of (e) ^15^N^13^C-alanine and (f) ^15^N^13^C tri-alanine.

* Denotes a significant difference (p<0.05) between the recovery in root biomass and shoot biomass.

*^1^: F_(2,7)_=45.75, p<0.001 *^2^: F_(2,7)_=21.30, p<0.001 *^3^: F_(2,7)_=20.93, p=0.001

*^4^: F_(2,8)_=51.45, p=0.001 *^5^: F_(2,7)_=25.74, p=0.001

ᵻ Denotes a significant difference (p<0.05) between the recovery in total plant biomass and microbial biomass.

ᵻ^1^: F_(1,3)_=19.87, p-0.021 ᵻ^2^: F_(1,4)_=28.17, p=0.006 ᵻ^3^: F_(1,4)_=35.30, p=0.004

ᵻ^4^: F_(1,3)_=103.22, p=0.002 ᵻ^5^: F_(1,3)_=46.06, p=0.007 ᵻ^6^: F_(1,3)_=68.12, p=0.004

ᵻ^7^: F_(1,4)_=108.88, p<0.001 ᵻ^8^: F_(1,4)_=22.54, p=0.009 ᵻ^9^: F_(1,4)_=6.00, p=0.014

ᵻ^10^: F_(1,3)_=60.71, p=0.004 ᵻ^11^: F_(1,4)_=6.82, p=0.009 ᵻ^12^: F_(1,4)_=6.82, p=0.009

Supplementary Figure 4

y= 0.4334x - 0.743

R^2^= 0.9555

y= 57.297x + 173.33

R^2^= 0.8238

y= 5.3811x + 130.97

R^2^= 0.7957

Significant regressions of nmol ^13^C excess g^-1^ DW vs. nmol ^15^N excess g^-1^ DW for (a) extracts of microbial biomass (black circles) in the high productivity grassland following labelled alanine addition, (b) root material (black circles) in the low productivity grassland following labelled tri-alanine addition, and (c) *Luzula* sp. shoot material (black circles) in the low productivity grassland following labelled tri-alanine addition. White circles indicate the ratio of ^15^N and ^13^C within (a) ^15^N^13^C-labelled alanine, and (b and c) ^15^N^13^C-labelled tri-alanine.
